# Supplementary material for: Interpolating numerically exact many-body wave functions for accelerated molecular dynamics
Source: Nat Commun. 2025 Feb 26;16:2005. doi: 10.1038/s41467-025-57134-9 (PMC11865551; doi:10.1038/s41467-025-57134-9)
Supplement: Supplementary file 1 — Supplementary information [file 41467_2025_57134_MOESM1_ESM.pdf]

Supplementary information for 'Interpolating numerically exact many-body wave functions for accelerated molecular dynamics'

Yannic Rath[1,2*] and George H. Booth[2*]

[1]National Physical Laboratory, Teddington, TW11 0LW, United Kingdom.
[2]Department of Physics and Thomas Young Centre, King's College London, Strand, London, WC2R 2LS, United Kingdom.

*Corresponding author(s). E-mail(s): yannic.rath@npl.co.uk; george.booth@kcl.ac.uk;

Supplementary information

Accuracy of the potential energy surface for hydrogen chains

In Fig. S1 we show the difference between the energy predicted by the eigenvector continuation and the reference DMRG energy for the converged MD trajectory of the 30-atom hydrogen system of the main text. It can be seen that, even though there is no prior knowledge about the targeted trajectory, the on-the-fly dataset generation for the molecular dynamics simulation achieves a potential energy surface resolved to roughly a few millihartree along the whole trajectory. The fluctuations in this error, often to close to zero, reflect the distance of the configuration explored in the trajectory to the training geometries. Since the training data is essentially exact, the inferred model must also be necessarily exact at these points, leading to the decreases to zero in the log of the error near these parts of the phase space in the trajectory.

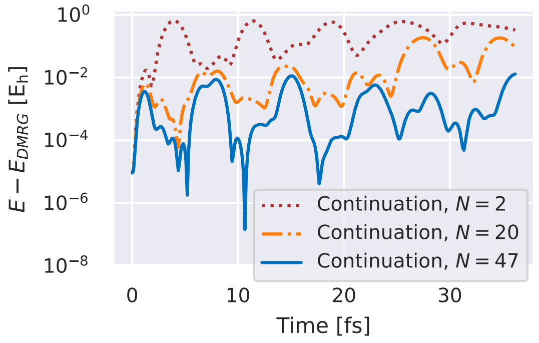

Fig. S1 Energy difference between reference density matrix renormalization group (DMRG) energies and the DMRG-trained eigenvector continuation for the trajectory of the 30-atom hydrogen chain (corresponding to Fig. 5 of the main text), with $N = 2$, $N = 20$, and $N = 47$ training data points. Source data are provided as a Source Data file.

Validation of training for thermalized Zundel cation ensembles

While it is possible to exploit the variationality of the approach to demonstrate convergence via systematic lowering of the energy across the trajectory, for the dynamics of the NVT ensemble of nuclear trajectories of the Zundel system (main text Fig. 6), we validate the accuracy of the inference via comparison to additional explicit DMRG calculations along the generated trajectories that are not in the training set. We select the geometries for this validation data set by sampling a

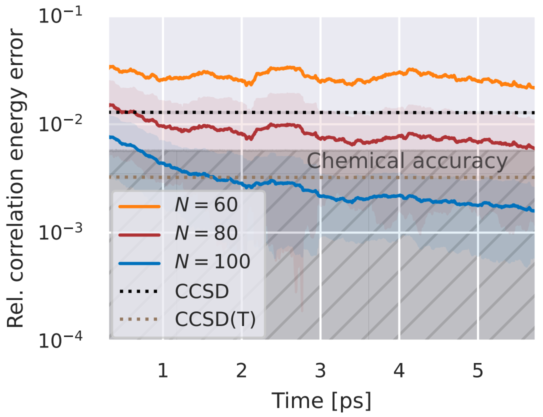

Fig. S2 Relative correlation energy error for the ensemble of Zundel cation trajectories with different numbers of training geometries (N), taken from a sub-sampling of 1000 representative geometries from equidistant times along the final $N = 100$ trajectory. This quantifies the test error across the trajectories against additional reference density matrix renormalization group calculations not used in their training. The plot depicts the running average over 10 out of the total of 1000 test geometries, with the corresponding standard deviation highlighted by the shaded areas, and 'chemical accuracy' denoted by the gray shaded area – an accuracy for which the $N = 100$ model clearly surpasses for the thermalized dynamics. Source data are provided as a Source Data file.

total of 1000 configurations from all the trajectories (generated from $N = 100$ training data points) at equidistant time intervals along the evolution.

Figure S2 shows the relative correlation energy error from the prediction of the continuation model with $N = 60, 80$ and 100 training data sizes, compared to 1000 exact reference points obtained via DMRG for this validation set over the trajectory. The solid line represents a running average over ten test geometries, with additional shading indicating the standard deviation. The consistent reduction in the energy error as more data points are used for the prediction is evident. For a training set with $N = 100$ configurations, we obtain a mean correlation energy error below chemical accuracy for the thermalized system, also demonstrating an improved accuracy compared to both CCSD and CCSD(T), increasing our confidence in the fidelity of the predicted thermodynamic quantities.

Thermalized Zundel cation dipole moment

In addition to the statistical convergence of the oxygen-to-hydrogen distance for the thermalized ensemble, we additionally show the convergence of an electronic quantity of interest. The main panel of Fig. S3 shows the averaged norm of the dipole moment over the thermalized ensemble of trajectories (with respect to the center of mass of the system) as a function of the propagation time, with the inset displaying the associated thermalized distribution function at the final time. As a response property, this is likely to be a more sensitive quantity with respect to the quality of the electronic structure over the trajectory. While the DMRG-trained interpolation (with $N = 100$) gives a flatter thermalized distribution function for the oxygen-hydrogen distance compared to DFT (see Fig. 6 of the main text), we find that the magnitude of the dipole moment is reduced compared to DFT (both with CAM-B3LYP and PBE exchange correlation functionals) and CCSD, both for the final thermalized ensemble, and for the full trajectory. Density functional theory in particular gives a flatter and more skewed thermalized distribution compared to both CCSD and the continuation.

Zundel cation energy surface convergence for high-energy trajectory

Figure S4 demonstrates the convergence of the DMRG-trained potential energy surface (PES) underpinning the high-energy Zundel cation NVE trajectory shown in Fig. 7 of the main text. In the top panel, we show the final $N = 84$ inferred electronic energy along the trajectory, compared to approximate Hartree–Fock (HF) and coupled cluster with single and double excitations (CCSD). The obtained values improve significantly upon single point energy estimates from Hartree-Fock, and are generally comparible with CCSD over the whole simulation, noting that CCSD is not a variational method and therefore can be above or below the exact energy. Nevertheless, we expect CCSD to be generally an accurate method to describe this system over many of the geometries visited. However, it is significant that for approximately 40 geometries along the $N = 84$ trajectory, CCSD could not converge with reasonable simulation

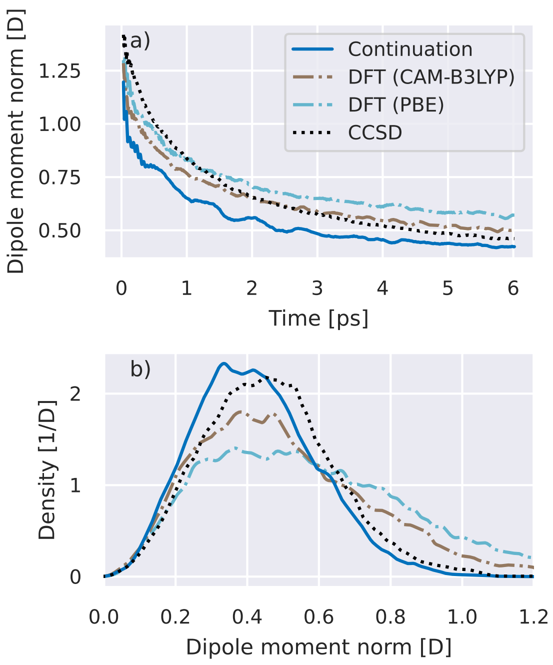

Fig. S3 Dipole moment norm as a function of propagation time averaged over the ensemble of 500 trajectories for the same setup as discussed in Fig. 6 of the main text. Panel a) depicts a running average over 100 propagation timesteps, and panel b) shows the thermal distribution function obtained via a kernel density analysis with Gaussian smearing with $\sigma = 0.01\,\mathrm{D}$ over the geometries of the last 100 iterations. Continuation results correspond to the $N = 100$ interpolation model. Source data are provided as a Source Data file.

and convergence parameters, indicating that more strongly correlated electronic structure is found along the trajectory. In contrast, the eigenvector continuation, which lacks any self-consistent or iterative aspects in inferring the electronic structure for each geometry (even at a mean-field level), can be run in an entirely robust and black-box fashion, with the ability to describe strongly correlated geometries only dependent on the solver used for the training.

In the center panel of the figure we highlight the convergence of the PES as a function of the number of training geometries used, showing the average electronic energy over the final trajectory for different numbers of training points (shifting the energies such that the $N = 84$ point has a zero average PES). While the variational character of the continuation guarantees a monotonic decrease of this average potential energy as more training configurations are added, various flat regions can be observed, indicating training configurations not contributing to a substantial improvement to the potential energy of the final trajectory. This highlights the potential for further improvements to the data selection scheme in order to identify the most appropriate training configurations and further accelerate the convergence of the PES with training points.

Finally, the bottom panel of Fig. S4 shows the energy at the 84 training configurations used to train the model in the final converged MD simulation. At a number of these training geometries, the HF and/or CCSD were not able to fully converge, and these points have been omitted. While optimizing convergence parameters may enable convergence, this is potentially a flag for stronger correlation effects at these points, where the DMRG-trained continuation would be more valuable and HF and CCSD unsuitable. Furthermore, there are clear indications of at least 13 training points where even the DMRG training data was not able to correctly converge, likely due to getting stuck in local minima in the DMRG sweep algorithm that optimizes the MPS.

This also highlights the lack of 'black-box' character for these highly accurate wave function based electronic structure methods, which means that much care is needed if they were to be applied to MD on their own. Likely the convergence of the continuation with respect to training points would have been improved had these been fully converged, however it is noteworthy that this does not substantially impact upon the quality of the inference of the eigenvector continuation at these training points, which is found to be substantially more accurate (variationally lower) than their corresponding training point at these points even where the optimization of the training wave function failed. This is analogous to the robustness of the interpolation discussed in the context of Fig. 4 of the main text for CASCI training in the presence of discontinuities. Similarly, the surface for the MD trajectory necessarily remains smooth and the gradients well-behaved. We note that the nature of the active data selection means that the molecular geometries of these training points are unlikely to actually feature in the final MD trajectory, since each time the data set is enlarged, the PES changes and the MD follows a different path. In the final MD simulation, the HF does converge

for all points along the trajectory, and CCSD with default optimization parameters fails $\sim 4\%$ of the time.

We note finally that the rationale for selection of additional data points via the Hamiltonian metric of Eq. 16 in the main text builds upon the assumption that data points add the correct training state to the dataset and therefore significantly improve the potential energy surface at that geometry. Adding data points for which an appropriately converged solution to the ground state was not obtained (in this case with DMRG) can thus negatively affect the performance of the data selection for subsequent runs and the rate of convergence of the method to exactness. Improving the convergence of the molecular dynamics simulation even in limits where the data set comprises inaccurate solutions to the electronic structure problem remains a subject of future research.

Nuclear forces for eigenvector continuation

Based on the potential energy estimate $E(\mathbf{R})$ for a nuclear configuration $\mathbf{R}$, the force can be defined as

$$F(\mathbf{R}) = -\frac{\partial E(\mathbf{R})}{\partial \mathbf{R}} = -\left(\frac{\partial E_{\text{nuc}}(\mathbf{R})}{\partial \mathbf{R}} + \frac{\partial E_{\text{el}}(\mathbf{R})}{\partial \mathbf{R}}\right), \quad (1)$$

which as shown can be split up into the electronic and nuclear contributions to this force. Focusing on the electronic contribution from the eigenvector continuation (and dropping the subscript for clarity), we apply the Hellmann-Feynman theorem [3], within the restricted subspace spanned by the training states, giving

$$\frac{\partial E(\mathbf{R})}{\partial \mathbf{R}} = \frac{\partial}{\partial \mathbf{R}}\left(\boldsymbol{\psi}^{\dagger}\, \boldsymbol{\mathcal{H}}\, \boldsymbol{\psi}\right) = \boldsymbol{\psi}^{\dagger}\, \frac{\partial \boldsymbol{\mathcal{H}}}{\partial \mathbf{R}}\, \boldsymbol{\psi}, \quad (2)$$

where $\boldsymbol{\psi}$ is the inferred ground-state eigenfunction from the diagonalization of the Hamiltonian in the many-body basis of the fixed training states (Eq. 2 of the main text). Substituting in the definition of the Hamiltonian from Eq. 5, similar to the derivation in Ref. [10], we obtain

$$\frac{\partial E(\mathbf{R})}{\partial \mathbf{R}} = \sum_{ijkl} \frac{\partial K_{ijkl}(\mathbf{R})}{\partial \mathbf{R}}\, \Gamma^{ijkl} \quad (3)$$

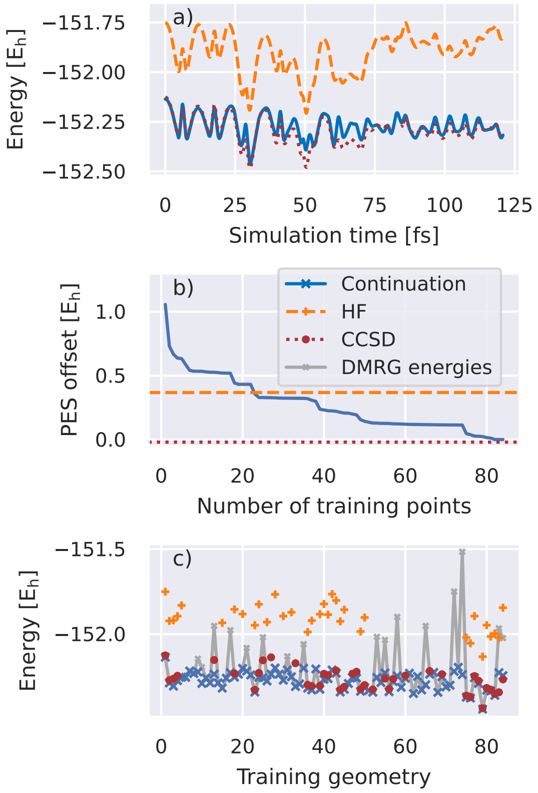

Fig. S4 Characteristics of potential energy surfaces and single point training energy estimates for the simulation of the water-hydronium reaction from the eigenvector continuation (blue), Hartree-Fock ('HF', orange), as well as coupled-cluster with singles and doubles ('CCSD', red). The top panel a) shows the potential energy for these methods as a function of time over the $N = 84$ converged trajectory. Panel b) shows the average energy over the final trajectory which systematically converges as more training data points are included in the eigenvector continuation. Panel c) shows the single point energy estimates for each of the $N = 84$ training geometries. Blue crosses denote the energy of the density matrix renormalization group (DMRG) trained eigenvector continuation at the training geometries, the gray line gives the single point energy estimates from the individual DMRG calculations used for the training points (noting the spikes corresponding to poor DMRG convergence), while red dots and orange crosses show the CCSD and HF values at the training geometries which were able to be converged along the trajectory with default parameters. Source data are provided as a Source Data file.

where Γ^{ijkl} is the two-electron reduced density matrix of the inferred state,

$$\Gamma^{ijkl} = \sum_{a,b}^{N} \psi_a^{*}\, \Gamma_{ab}^{ijkl}\, \psi_b. \quad (4)$$

The reduced Hamiltonian gradient, $\frac{\partial K_{ijkl}(\mathbf{R})}{\partial \mathbf{R}}$, is obtained according to

$$\frac{\partial K_{ijkl}(\mathbf{R})}{\partial \mathbf{R}} = \frac{1}{2}\frac{\partial h^{(2)}_{ijkl}(\mathbf{R})}{\partial \mathbf{R}} + \frac{1}{2(N_{\text{elec}}-1)}\left(\delta_{jl}\frac{\partial h^{(1)}_{ik}(\mathbf{R})}{\partial \mathbf{R}} + \delta_{ik}\frac{\partial h^{(1)}_{jl}(\mathbf{R})}{\partial \mathbf{R}}\right) \quad (5)$$

The evaluation of the the one- and two-electron integral gradients in the SAO basis requires the derivative of the transformation $\mathbf{Z}(\mathbf{R})$ from the atomic orbitals to the SAO basis (a Pulay-like force contribution), as well as the gradients of the integrals between atomic orbitals. For the one-electron integral derivative, we obtain

$$\frac{\partial h^{(1)}_{ij}}{\partial \mathbf{R}} = \sum_{\alpha\beta} Z_{\alpha i}\, Z_{\beta j}\, \frac{\partial \tilde{h}^{(1)}_{\alpha\beta}}{\partial \mathbf{R}} + \sum_{\alpha\beta} \frac{\partial Z_{\alpha i}}{\partial \mathbf{R}}\, Z_{\beta j}\, \tilde{h}^{(1)}_{\alpha\beta} + \sum_{\alpha\beta} \frac{\partial Z_{\beta j}}{\partial \mathbf{R}}\, Z_{\alpha i}\, \tilde{h}^{(1)}_{\alpha\beta}, \quad (6)$$

where greek indices refer to atomic orbitals, latin indices to SAO, and $\tilde{h}^{(1)}_{\alpha\beta}$ denotes the one-electron integral in the atomic orbital basis. The derivatives of the integrals over the atomic orbitals, are readily accessible from standard libraries, and we utilize the implementation in the *Libcint* library [7] as bundled in the *PySCF* package [8, 9].

The gradient of the SAO transformation, $\frac{\partial Z_{\alpha i}}{\partial \mathbf{R}}$, follows directly from the definition of the Löwdin orthogonalization, and application of the chain rule. This gives

$$\frac{\partial Z_{\alpha i}}{\partial \mathbf{R}} = \sum_{\beta\gamma} \frac{\partial S_{\beta\gamma}}{\partial \mathbf{R}} \frac{\partial (\mathbf{S}^{-1/2})_{i\alpha}}{\partial S_{\beta\gamma}}. \quad (7)$$

In addition to the derivative of the overlap matrix between atomic orbitals, this requires the evaluation of the derivative of the inverse square root matrix elements with respect to the overlap elements, $\frac{\partial (\mathbf{S}^{-1/2})_{i\alpha}}{\partial S_{\beta\gamma}}$. To evaluate this derivative, we consider the spectral decomposition of $\mathbf{S}$ according to

$$\mathbf{S} = \mathbf{T}\,\mathbf{s}\,\mathbf{T}^{\dagger}, \quad (8)$$

from which we obtain

$$\frac{\partial \mathbf{S}^{-1/2}}{\partial S_{\beta\gamma}} = \frac{\partial \mathbf{T}}{\partial S_{\beta\gamma}}\,\mathbf{s}^{-1/2}\,\mathbf{T}^{\dagger} + \mathbf{T}\,\frac{\partial \mathbf{s}^{-1/2}}{\partial S_{\beta\gamma}}\,\mathbf{T}^{\dagger} + \mathbf{T}\,\mathbf{s}^{-1/2}\,\frac{\partial \mathbf{T}^{\dagger}}{\partial S_{\beta\gamma}}. \quad (9)$$

The eigenvector and eigenvalue derivatives can be evaluated via first order perturbation theory of each element of $S_{\beta\gamma}$ and evaluating the derivative at vanishing perturbation strength [1]. The chain rule then provides

$$\frac{\partial s_i^{-1/2}}{\partial S_{\beta\gamma}} = -\frac{1}{2}\, s_i^{-3/2}\, \frac{\partial s_i}{\partial S_{\beta\gamma}} = -\frac{1}{2}\, s_i^{-3/2}\, T^{*}_{\beta i}\, T_{\gamma i}, \quad (10)$$

and we obtain for the derivative of the eigenvector coefficients

$$\frac{\partial T_{\alpha i}}{\partial S_{\beta\gamma}} = -\sum_{j\neq i} \frac{T^{*}_{\beta j}\, T_{\gamma i}\, T_{\alpha j}}{s_j - s_i}. \quad (11)$$

The sum in the above expression runs over all eigenstates except i. Additional care needs to be taken if eigenvalue degeneracies are present in Eq. (8), in which case we appeal to standard degenerate perturbation theory [1], ensuring we rotate the eigenvectors in each degenerate subspace such that the applied perturbation in this degenerate subspace is diagonal. In this case, Eq. (11) becomes valid by only summing over all eigenstates which have a distinct eigenvalue from s_i.

Details of GAP model predictions

Within the application of the GAP framework, the potential energy for a test geometry is predicted based on a kernel model according to

$$E(\mathbf{R}) = \sum_{a=1}^{N} w_a\, k(\mathbf{R}, \mathbf{R}^{(a)}), \quad (12)$$

where the weights w_a may be interpreted as learned parameters of the model, and the kernel function k defines a similarity measure between nuclear geometries.

Without fully optimizing the various design choices, we follow standard approaches to construct a suitable kernel function within the GAP framework based on the idea of a smooth overlap of atomic positions (SOAP) [2]. This representation defines a set of features for each atom in

the system based on its local atomic environment represented in a basis of spherical harmonics together with radial basis functions automatically incorporating important symmetries into the representation. We utilize the implementation of the SOAP features from the *dscribe* library [5], implementing the SOAP features as described in Ref. [2]. Without additional hyperparameter tuning, we constructed the SOAP features in our tests by going up to 20th order in the spherical harmonics and 10th order in the radial basis functions, and chose a radial cutoff of 10 Å.

Based on these SOAP features, we construct an averaged kernel function [2], given as the average of scalar products between the SOAP feature vectors over all pairs of atoms in the system. Denoting the vector of SOAP features for the i-indexed atom as $\mathbf{p}(\mathbf{R}_i)$, this kernel, appropriately normalized, is thus given as

$$k(\mathbf{R}, \mathbf{R}') = \frac{\sum_{ij} \mathbf{p}(\mathbf{R}_i) \cdot \mathbf{p}(\mathbf{R}'_j)}{\sqrt{\mathcal{N}(\mathbf{R}) \times \mathcal{N}(\mathbf{R}')}}, \tag{13}$$

with a normalization constant

$$\mathcal{N}(\mathbf{R}) = \sum_{ij} \mathbf{p}(\mathbf{R}_i) \cdot \mathbf{p}(\mathbf{R}_j). \tag{14}$$

Following standard Gaussian process regression techniques [6], the weights of the model are obtained by inversion of the kernel matrix according to

$$\mathbf{w} = (\mathbf{K} + \sigma^2 \mathbb{1})^{-1} \mathbf{E}. \tag{15}$$

Here, $\mathbf{w}$ denotes the vector of weights for all data points, $\mathbf{K}$ is the matrix of pairwise kernel values between the training configurations, and $\mathbf{E}$ is a vector comprising the training energies. For the results shown in Fig. 2 of the main text, we set the additional noise parameter σ^2, effectively regularizing the fit, to a small fixed value of $\sigma^2 = 10^{-15}$, without additional optimization.

While it is possible to train GAP models also on force data to improve the prediction [4], here we simply estimate the force by differentiating through the potential energy predictor according to Eq. (12). The force prediction is therefore obtained as

$$F(\mathbf{R}) = -\sum_{a=1}^{N} w_a \frac{\partial k(\mathbf{R}, \mathbf{R}^{(a)})}{\partial \mathbf{R}}, \tag{16}$$

with the kernel function as defined in Eq. (13). It should be noted that the SOAP features used for the kernel are atom-centered, and therefore the derivative with respect to these basis functions needs to be taken into account when evaluating the gradient, as implemented in the *dscribe* library [5].

References

[1] Bassam Bamieh. A tutorial on matrix perturbation theory (using compact matrix notation), 2022.

[2] Sandip De, Albert P. Bartók, Gábor Csányi, and Michele Ceriotti. Comparing molecules and solids across structural and alchemical space. *Phys. Chem. Chem. Phys.*, 18:13754–13769, 2016.

[3] R. P. Feynman. Forces in molecules. *Phys. Rev.*, 56:340–343, Aug 1939.

[4] Aldo Glielmo, Peter Sollich, and Alessandro De Vita. Accurate interatomic force fields via machine learning with covariant kernels. *Phys. Rev. B*, 95:214302, Jun 2017.

[5] Lauri Himanen, Marc O.J. Jäger, Eiaki V. Morooka, Filippo Federici Canova, Yashasvi S. Ranawat, David Z. Gao, Patrick Rinke, and Adam S. Foster. Dscribe: Library of descriptors for machine learning in materials science. *Comput. Phys. Commun.*, 247:106949, 2020.

[6] Carl Edward Rasmussen, Christopher KI Williams, et al. *Gaussian processes for machine learning*, volume 1. Springer, 2006.

[7] Qiming Sun. Libcint: An efficient general integral library for gaussian basis functions. *J. Comput. Chem.*, 36(22):1664–1671, 2015.

[8] Qiming Sun, Timothy C. Berkelbach, Nick S. Blunt, George H. Booth, Sheng Guo, Zhendong Li, Junzi Liu, James D. McClain,

Elvira R. Sayfutyarova, Sandeep Sharma, Sebastian Wouters, and Garnet Kin-Lic Chan. Pyscf: the python-based simulations of chemistry framework. *WIREs Comput. Mol. Sci.*, 8(1):e1340, 2018.

[9] Qiming Sun, Xing Zhang, Samragni Banerjee, Peng Bao, Marc Barbry, Nick S. Blunt, Nikolay A. Bogdanov, George H. Booth, Jia Chen, Zhi-Hao Cui, Janus J. Eriksen, Yang Gao, Sheng Guo, Jan Hermann, Matthew R. Hermes, Kevin Koh, Peter Koval, Susi Lehtola, Zhendong Li, Junzi Liu, Narbe Mardirossian, James D. McClain, Mario Motta, Bastien Mussard, Hung Q. Pham, Artem Pulkin, Wirawan Purwanto, Paul J. Robinson, Enrico Ronca, Elvira R. Sayfutyarova, Maximilian Scheurer, Henry F. Schurkus, James E. T. Smith, Chong Sun, Shi-Ning Sun, Shiv Upadhyay, Lucas K. Wagner, Xiao Wang, Alec White, James Daniel Whitfield, Mark J. Williamson, Sebastian Wouters, Jun Yang, Jason M. Yu, Tianyu Zhu, Timothy C. Berkelbach, Sandeep Sharma, Alexander Yu. Sokolov, and Garnet Kin-Lic Chan. Recent developments in the PySCF program package. *J. Chem. Phys.*, 153(2):024109, 07 2020.

[10] Robert E. Thomas, Daniel Opalka, Catherine Overy, Peter J. Knowles, Ali Alavi, and George H. Booth. Analytic nuclear forces and molecular properties from full configuration interaction quantum Monte Carlo. *J. Chem. Phys.*, 143(5):054108, 08 2015.
