# Supplementary figures and images for: Interpolating numerically exact many-body wave functions for accelerated molecular dynamics
Source: Nat Commun. 2025 Feb 26;16:2005. doi: 10.1038/s41467-025-57134-9 (PMC11865551; doi:10.1038/s41467-025-57134-9)

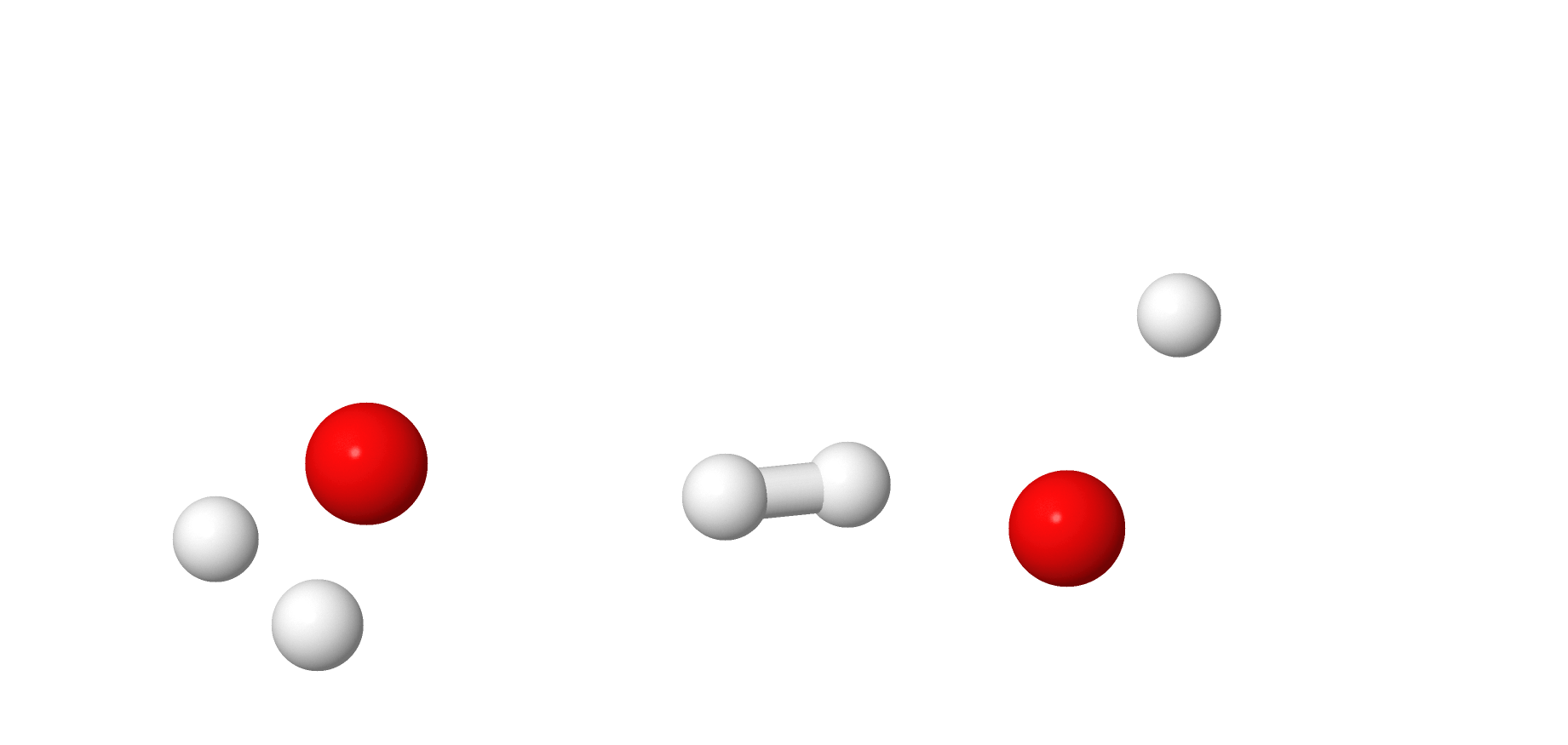

Supplement: Supplementary file 3 — Source data [file 41467_2025_57134_MOESM3_ESM.zip › Source Data/fig_7/geometry_snapshot_1.png]

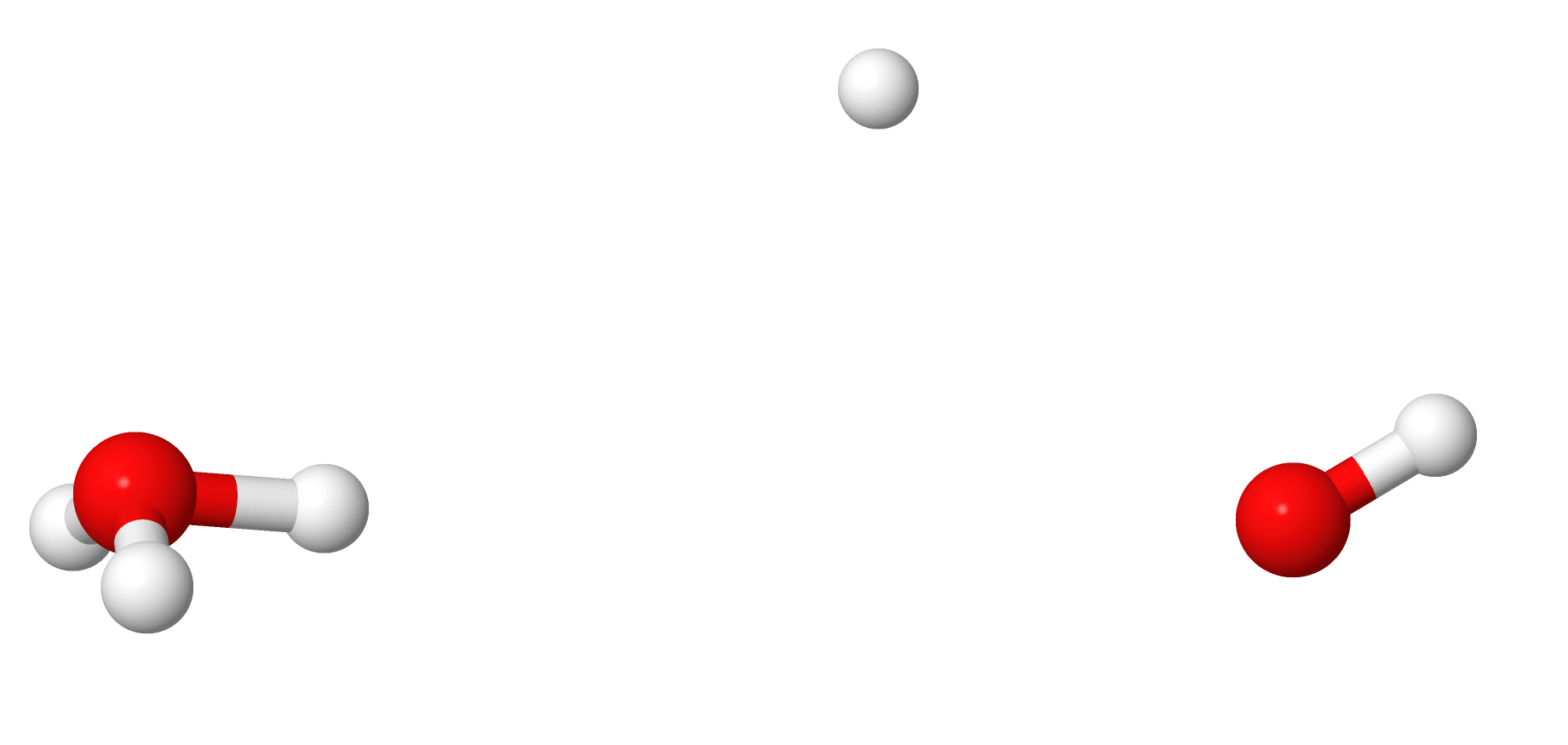

Supplement: Supplementary file 3 — Source data [file 41467_2025_57134_MOESM3_ESM.zip › Source Data/fig_7/geometry_snapshot_1000.png]

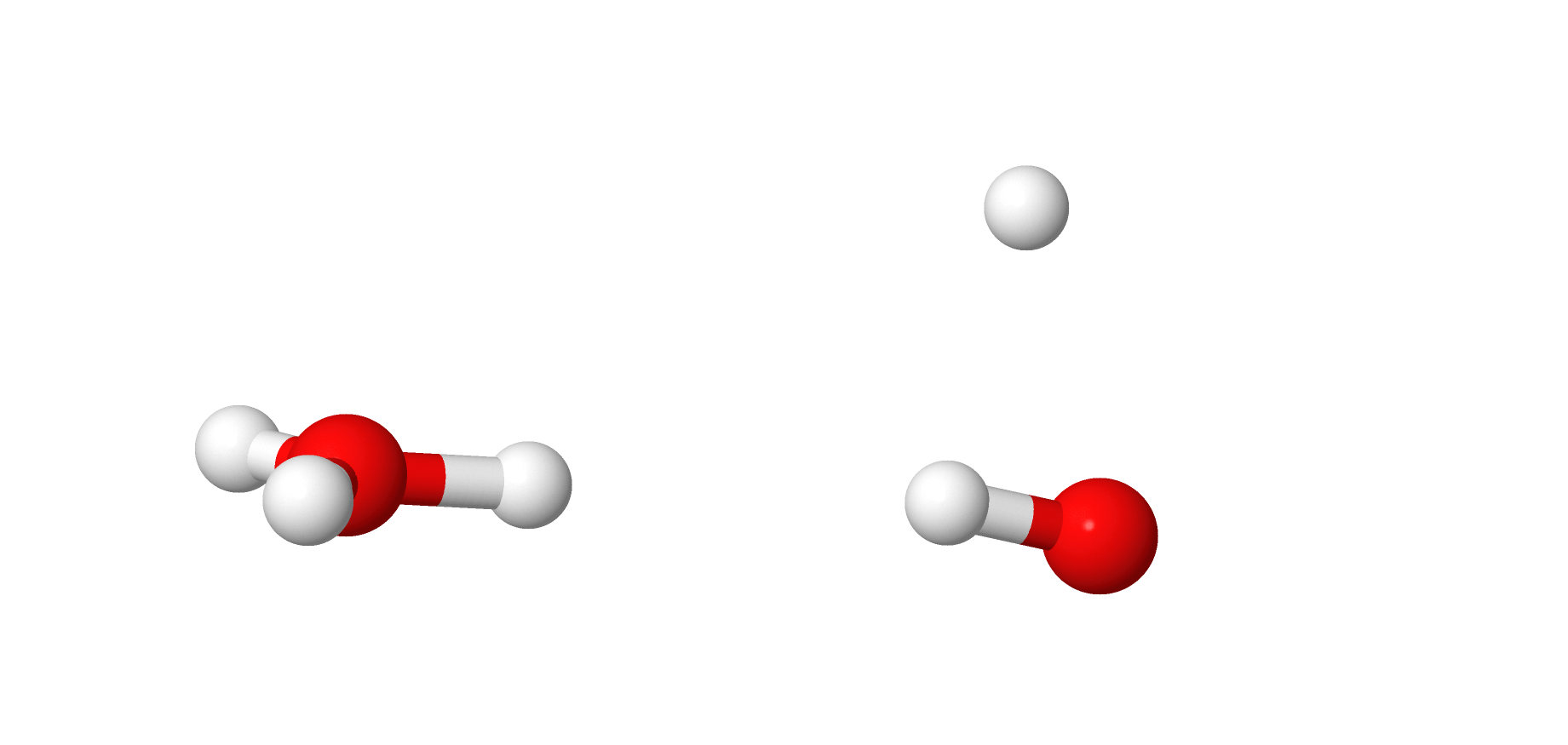

Supplement: Supplementary file 3 — Source data [file 41467_2025_57134_MOESM3_ESM.zip › Source Data/fig_7/geometry_snapshot_334.png]

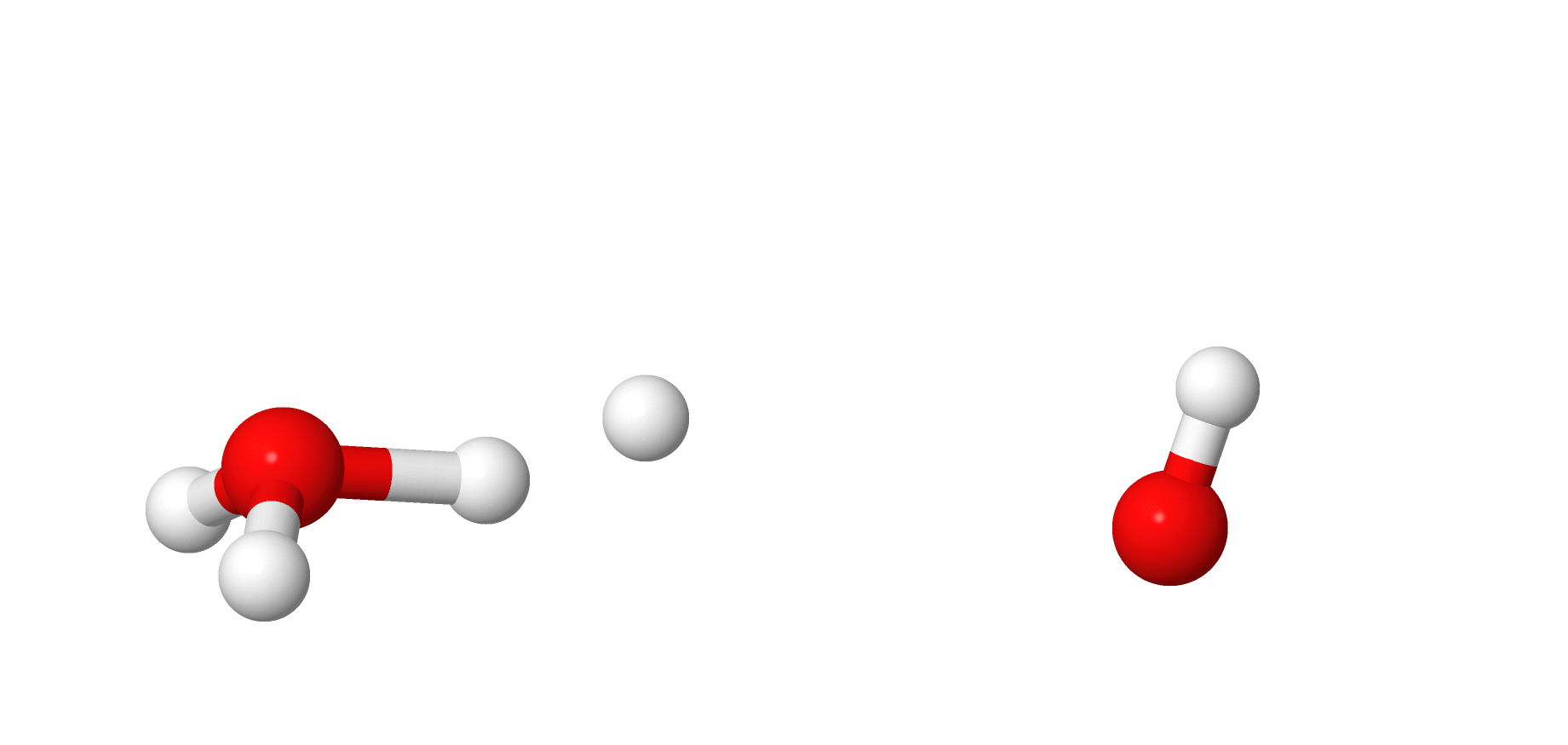

Supplement: Supplementary file 3 — Source data [file 41467_2025_57134_MOESM3_ESM.zip › Source Data/fig_7/geometry_snapshot_667.png]
